# Enterectomy Contrasted with Artificial Anus

**Published:** 1902-08-16

**Authors:** 


### ENTERECTOMY CONTRASTED WITH ARTIFICIAL ANUS.

There are certain cases in the treatment of which the production of an artificial anus has seemed to many surgeons to give the best chance of life. But even life when coupled with an artificial anus is in some cases a blessing very much disguised, while it must be remembered that the production of such an opening of the gut on to the surface of the skin has by no means always availed to avert death. Discussing the treatment of such cases Mr. Arthur Barker[1] urges that in many instances it is better to excise the affected portion of bowel, and join the cut ends together, than to make an artificial anus—better, in fact, to make the bowel empty into bowel than to allow it to empty itself on to the surface of the skin; and he maintains that, with the improved technique now at our disposal, the mortality of such a proceeding is probably no greater, possibly even less, than that of the older method, while as a result, instead of the patient being left exposed to all the miseries which must attend the discharge of fæces in an abnormal position, he is restored, when he does recover, to a state of comfort. A glance at the past will, he says, convince anyone that gangrenous strangulation treated by artificial anus had a high mortality, to say nothing of the distress and misery associated with discharge of liquid fæces and semi-starvation, if not total starvation. Again, although colotomy for malignant stricture is a most valuable operation, and besides prolonging life remedies an otherwise most distressing state of things, it does so often at the expense of considerable discomfort, and leaves the patient with the feeling that the end must still be kept in view. But in regard to enterectomy we now know that primary excision of gangrenous gut is not only justifiable but in proper cases offers the best chance as to life; while in neoplasm of the bowel the growth can be freely removed, and the continuity of the tube restored, with but moderate risk; and further, that in many cases in which, owing to delay, the growth has relations which render its removal impossible, the continuity of the bowel can nevertheless be renewed by short circuit. In illustration of these propositions he gives an admirable series of cases which he has treated on the principle laid down.

[1] Brit. Med. Jour., June 28.

